# Scalp spindles are associated with widespread intracranial activity with unexpectedly low synchrony

**DOI:** 10.1016/j.neuroimage.2014.10.048

**Published:** 2015-01-15

**Authors:** Birgit Frauscher, Nicolás von Ellenrieder, François Dubeau, Jean Gotman

**Affiliations:** a Montreal Neurological Institute and Hospital, McGill University, 3801 University Street, Montreal H3A 2B4, Canada; b Innsbruck Medical University, Department of Neurology, Anichstrasse 35, A-6020 Innsbruck, Austria; c CONICET–LEICI, Universidad Nacional de La Plata, Calle 1 y 47, La Plata B1900TAG, Argentina

**Keywords:** Electroencephalography, Hippocampus, Human, Insula, Sleep physiology, Non-rapid eye movement sleep

## Abstract

In humans, the knowledge of intracranial correlates of spindles is mainly gathered from noninvasive neurophysiologic and functional imaging studies which provide an indirect estimate of neuronal intracranial activity. This potential limitation can be overcome by intracranial electroencephalography used in presurgical epilepsy evaluation. We investigated the intracranial correlates of scalp spindles using combined scalp and intracerebral depth electrodes covering the frontal, parietal and temporal neocortex, and the scalp and intracranial correlates of hippocampal and insula spindles in 35 pre-surgical epilepsy patients. Spindles in the scalp were accompanied by widespread cortical increases in sigma band energy (10–16 Hz): the highest percentages were observed in the frontoparietal lateral and mesial cortex, whereas in temporal lateral and mesial structures only a low or no simultaneous increase was present. This intracranial involvement during scalp spindles showed no consistent pattern, and exhibited unexpectedly low synchrony across brain regions. Hippocampal spindles were shorter and spatially restricted with a low synchrony even within the temporal lobe. Similar results were found for the insula. We suggest that the generation of spindles is under a high local cortical influence contributing to the concept of sleep as a local phenomenon and challenging the notion of spindles as widespread synchronous oscillations.

## Highlights

• Spindles in the scalp are accompanied by widespread cortical spindle activity.
• This activity is predominantly present in the frontoparietal lateral and mesial cortex.
• The intracranial involvement during scalp spindles shows no consistent pattern.
• The synchrony of spindles is unexpectedly low across different brain regions.
• Hippocampal spindles were shorter and occurred mostly not at time of scalp spindles.

## Introduction

Spindles are distinct electroencephalographic (EEG) events which are the hallmark of non-rapid eye movement sleep stage 2. They are characterized by waxing and waning oscillations with a frequency between 10 and 16 Hz and duration between 0.5 and 2 s (Loomis et al., 1935; Gibbs and Gibbs, 1950; Jankel and Niedermeyer, 1985). Recent research points to the importance of spindles for memory consolidation, cortical development, and sleep stability (Khazipov et al., 2004; Schabus et al., 2004; Dang-Vu et al., 2010a; Fogel and Smith, 2011).

The “classical” thalamic model of spindle generation suggests that spindles are generated in the reticular thalamic nucleus, triggered by the depolarizing phase of the cortical slow oscillation via corticothalamic pathways. After being transferred to the dorsal thalamus, they project back to the cortex following thalamo-cortical circuits (Fuentealba and Steriade, 2005; Steriade, 2006). In humans, knowledge on spindles and their source localization is gathered from EEG (Anderer et al., 2001; Kurth et al., 2010; del Felice et al., 2013), magnetoencephalography (MEG) (Nakasato et al., 1990; Manshanden et al., 2002; Ishii et al., 2003; Urakami, 2008; Gumenyuk et al., 2009; Dehghani et al., 2011) and functional imaging studies with positron emission tomography and functional magnetic resonance imaging (Hofle et al., 1997; Laufs et al., 2007; Schabus et al., 2007; Tyvaert et al., 2008; Andrade et al., 2011; Caporro et al., 2012). These studies demonstrate that there are at least two different cortical spindle generators with a maximal source activity in the frontal and parietal neocortex (Anderer et al., 2001; Gumenyuk et al., 2009). In addition, functional imaging studies revealed that at the time of scalp spindles, a signal change is present in the frontal and parietal cortex, and in the thalamus, limbic regions, precuneus and cingulate gyrus, which would be compatible with the suggested gating sensory function of spindles and their involvement in memory and learning (Dang-Vu et al., 2010b). The results of source localization with EEG and MEG are highly dependent on the algorithms used for solving the inverse problem, whereas functional imaging studies provide an indirect estimate of neuronal activity with a poor time resolution as they primarily target hemodynamic changes.

These limitations can be overcome by intracranial EEG used in the setting of the presurgical investigation of therapy-refractory epilepsy. Only two studies looked at characteristics of spindles in a small number of subjects and over different brain regions using intracerebral depth electrodes (Andrillon et al., 2011; Peter-Derex et al., 2012). Their major findings were that spindles occur across multiple brain regions, that spindle frequencies change along a caudo-rostral axis with faster spindles found in the centroparietal areas and slower spindles in the frontal regions and that 55% of spindles are more local than generalized when looking at overlapping activity in the spindle frequency band in different intracranial EEG channels (Andrillon et al., 2011; Peter-Derex et al., 2012). Because scalp EEG was not simultaneously analyzed during these intracerebral EEG studies, they do not inform us about what happens in different brain regions when a spindle is recorded on the scalp. This is of great interest as it provides us with direct information of intracerebral correlates of scalp spindles, and allows us to investigate synchronization between scalp spindle events and intracerebral spindles. Recent studies using simultaneous EEG/MEG revealed that MEG spindles have low spatial coherence and exhibit low correlation with EEG spindles (Dehghani et al., 2011). The authors speculated that multiple asynchronous sources as revealed by MEG may overlap sufficiently to appear synchronously in the EEG (Dehghani et al., 2011). This is still an object of controversy and can be addressed with combined scalp and intracranial EEG. We therefore investigated the intracranial correlates of scalp spindles using combined scalp–intracranial EEG. Special attention was paid to the analysis of synchrony of intracranial spindles at the time of scalp spindles. In addition, we were interested to examine the scalp correlates of spindles in the hippocampus and insula as examples of brain structures remote from the scalp.

## Material and methods

### Patient selection

Forty-nine consecutive patients with pharmacoresistant temporal or extra-temporal lobe epilepsy underwent continuous intracranial EEG recordings combined with scalp EEG at the Montreal Neurological Institute and Hospital between January 2010 and February 2014 for seizure foci identification and potential surgical treatment.

We included patients for whom we had at least one continuous whole night recording which was at least 72 h post-electrode implantation in order to avoid a potential influence of anesthesia on sleep as well as the acute effect of electrode implantation. Also, to be as close as possible to the study of sleep in healthy subjects, we selected brain regions and periods of recording that were as little as possible affected by the epileptic activity always recorded in these patients. Hence, a second inclusion criterion was that patients' recordings had at least one intracranial EEG channel with no or very rare epileptic discharges and no other abnormalities, and that these channels were not within lesional tissue as assessed with MRI (see “Selection of suitable intracranial EEG channels” section). Thirdly, we only included subjects in whom epileptic activity did not interfere with spindle scoring and who had at least 5 unambiguous spindles in the scalp EEG during the first sleep cycle. Choosing a different cut-off of for instance 10 spindles would have resulted in the same number of eligible EEG recordings. In fact, only 3 patients had fewer than 50 spindles in the first sleep cycle with a minimum number of 31 spindles (see Supplementary Table 1). The spindle events were marked during the first sleep cycle in order to follow the same methodology in all subjects, as density of slow wave activity changes across the different sleep cycles, and there is evidence (Andrillon et al., 2011) that spindle density and frequency are influenced by this change in slow wave activity. Exclusion criteria were the presence of a secondarily generalized seizure during the 12 h, or the presence of a partial seizure or asymptomatic EEG seizure during the 6 h prior to the evaluated night of the sleep.

Thirty-five of the 49 patients met these selection criteria. Demographic and clinical data are provided in Supplementary Table 1. Reasons for exclusion were: recordings having no channels with no or rare epileptic discharges (n = 4), no channels located outside lesional tissue (n = 2), lack of at least five unambiguous spindle events in the first sleep cycle (n = 2), presence of partial or asymptomatic EEG seizures within 6 h or generalized seizures within 12 h of sleep recordings (n = 4), absence of a sleep recording 72 h post-implantation (n = 1), or the presence of epileptic discharges in the scalp EEG channels interfering with the scoring of sleep spindles (n = 1). In patients with several full-night recordings, the first night after 72 h post-implantation was evaluated.

This study was approved by the Research Ethics Board of the Montreal Neurological Institute and Hospital. All patients signed a Research Ethics Board approved written informed consent.

### Intracranial and scalp EEG recording

**Fig. 1:**
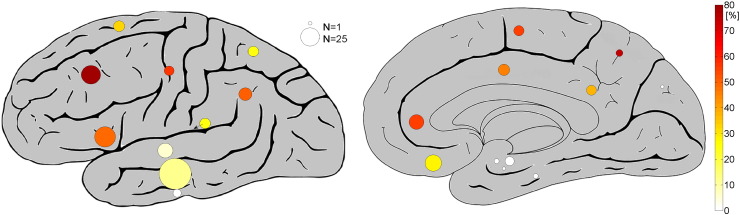
Illustration of the sampled brain regions and the percentages of intracranial sigma band activity at time of scalp spindles. The circles correspond to the investigated brain regions. The area of the circles is proportional to the number of investigated channels in each brain structure, and the color indicates the percentage of channels in which a statistically significant increase in sigma band energy (10–16 Hz) was observed during the scalp spindle intervals compared to the control intervals (with Holm–Bonferroni correction for multiple comparisons).

An average of seven multicontact depth electrodes (range, 4–12) was implanted stereotactically through an orthogonal or oblique approach using an image-guided system (ROSA robotic neuronavigation system or Medtronic Stealth neuronavigation system). In 21 patients, implantations consisted of electrodes manufactured on site (9 contacts, 0.5 to 1 mm in length and 5 mm apart); and in 14 patients, commercially available electrodes (DIXI Medical, France: 10 to 18 contacts, 2 mm in length and 1.5 mm apart) were used. The deepest contacts were targeting the mesial aspect of the lobe explored, and the most superficial ones were targeting the neocortex. The depth electrode montages were bipolar, from one contact to the neighboring contact. Epidural electrodes were additionally placed in eight patients. Electrode locations were determined by either post-implantation CT co-registered with a pre-implantation MRI using SPM 8 software (n = 15), post-implantation MRI (n = 14), or the information from the reconstructed planned position of the electrodes from the Neuronavigation System (n = 6). Subdermal thin wire electrodes (Ives, 2005; Young et al., 2006) were placed at the time of implantation at positions F3, F4, Fz, C3, C4, Cz, P3, P4, Pz (33 patients) or Fz, C3, C4, Cz, Pz (2 patients) allowing for sleep staging and spindle marking. Intracerebral electrode positions were tailored for each patient and depended on the clinical hypothesis on the location of the seizure generator and propagation of ictal discharge. Fig. 1 provides an overview of the number of channels investigated in the different cortical regions. Information on the localization of the electrode contacts of each patient is provided in Supplementary Fig. 1. The EEG signal was low-pass filtered at 500 Hz, and sampled at 2000 Hz. EEGs were recorded using the Harmonie EEG system (Stellate, Montreal, Canada).

### Selection of suitable intracranial EEG channels

Only bipolar intracranial channels showing no or very rare epileptic discharges as well as no other background abnormalities and artifacts were evaluated. Suitable channels were selected based on the first sleep cycle independently by two electrophysiologists. For channels for which the selection differed between the two raters, a consensus was found in a common scoring session. Ambiguous channels were discarded as we aimed to investigate physiological sleep patterns. Moreover, depth electrodes placed in lesions as revealed by MRI were excluded irrespective of whether they showed interictal epileptic discharges.

### Assessment of spindles in scalp EEG recordings

Sleep was scored manually in 30 s epochs (Berry et al., 2012). The identification of spindles was done visually by a sleep expert, as it presents the generally accepted approach to reliably mark scalp spindles, and the number of needed events is still reasonable for a visual approach (Warby et al., 2014). Spindles were identified during the first NREM sleep cycle by using a time scale of 30 mm/s in all scalp EEG channels (F3–C3, C3–P3, Fz–Cz, Cz–Pz, F4–C4, C4–P4). We did not use a traditional mastoid referential montage for the scoring of sleep, as this was not feasible for all patients due to the localization of the implanted depth electrodes and the risk of infection. Conventional sleep recording with the same electrodes using a bipolar and mastoid reference montage revealed in line with the existing literature (Werth et al., 1997) that most spindles seen in the bipolar montage can be also seen in a mastoid referential montage, usually with a 0.5 or 1 cycle difference in the onset of the spindle.

Spindle identification stopped after 50 spindles were marked, or after all available spindles were marked in the first sleep cycle in case there were fewer than 50. Spindles were defined according to their typical waxing and waning morphology, their frequency between 10 and 16 Hz and duration of at least 0.5 s. No filters were set for spindle detection and gain was adapted as best suitable. For each spindle, the peak of the first well-defined negative wave was marked using a band-pass filter between 5 and 20 Hz. In case of simultaneous spindles over different channels, the spindle with the highest amplitude was marked. For statistical analysis, for each spindle a non-spindle control segment lasting 1 s was selected automatically and manually checked in the same channel at a random interval of 5 to 6 s after the onset of the corresponding spindle. If the control segment overlapped another spindle, it was displaced to the closest spindle-free section.

### Assessment of spindles in hippocampus and insula

We also investigated the presence of sleep spindles in the hippocampus and insula as examples of brain regions remote from the scalp. The same methodology concerning spindle identification and choice of a non-spindle control interval was applied as for the scalp. The channels assessed for spindle analysis were selected based on their location and defined by post-implantation imaging. All channels in the hippocampus were analyzed as one group, and as for the scalp the onset of the spindle with the highest amplitude was marked. The same procedure was applied to the insula channels. The target number of spindles was, as for the scalp, either 50 or all available spindles in the first sleep cycle if there were fewer than 50.

### Data processing

For the analysis, we selected only the most medial and the most lateral bipolar channels of each electrode which were in the cerebral cortex as assessed via post-implantation imaging or reconstructed planned position of the electrodes (e.g. in case of an electrode with 9 contacts, contacts 1–2 and contacts 8–9 were evaluated if they were in the gray matter and showed no abnormal activity or artifacts). The recordings were made with a 2 kHz sampling rate, and the first data processing step was a down-sampling to 100 Hz (antialiasing zero phase FIR filter of order 160, cutoff frequency 25 Hz). All data processing was performed with Matlab R 2007b.

To decide which channels presented a change in the sigma band activity (10–16 Hz) at the same time as the marked scalp spindles, we tested whether the energy in the sigma band was the same in the spindle and control segments for each channel. First, the signal component in the sigma band was obtained, using a zero-phase FIR filter (order 160, 2 Hz transition bands, 70 dB attenuation on the low frequency stop-band and 50 dB on the high frequency stop band, 0.4% ripple in the pass band). From the filtered signal we computed the energy in the sigma band in each one second interval starting at the onset of the spindles and control epochs. The choice of the length of the epochs is somewhat arbitrary since spindle durations can vary. However, attempts to determine the duration of the spindles, visually or automatically, would also include arbitrary thresholds for the power or amplitude of the signal. A fixed duration has the advantage of easy reproducibility and this was the reason for our choice. The energy values from the epochs were log-transformed and a paired t-test was carried out for each channel. We adopted a significance level of p = 0.05, with a Holm–Bonferroni correction to account for multiple comparisons, considering the number of comparisons equal to the total number of channels. We also report the results with Holm–Bonferroni correction per region (i.e. considering the number of comparisons equal to the number of channels per region), and the uncorrected test results to illustrate the robustness of the data.

To determine the frequency of the spindles, we estimated their power spectral density (PSD). We defined the spindle frequency as the frequency at which the PSD attains a maximum in the sigma band. The spectrum of the spindle segments was computed in every channel. The squared magnitude of the Discrete Time Fourier Transform (DFT) was computed for segments of 1 s starting at the onset of each marked scalp spindle. Then, the DFT was averaged among all the spindles of each channel, to obtain the PSD.

The variation in time of the energy in the sigma band during spindles was analyzed in the different brain regions of interest. The time course of the energy content was computed in each channel with a zero-phase moving average FIR filter (length 0.25 s) applied to the squared amplitude of the signal in the sigma band. The averaged energy signal was obtained for the spindle intervals, and divided by the average signal in the control intervals. A single energy signal was obtained for each brain region by averaging the channels from the different patients.

To analyze the degree of synchrony between the scalp and intracranial EEG signals, we computed the cross-correlation. For each scalp spindle and control segment, we computed the normalized cross-correlation of the sigma band component of the signal in the scalp channel in which the event was marked and of all analyzed intracranial channels. The normalized cross correlation function was computed for a segment of 0.5 s starting at the onset of the marked events. The normalized cross-correlation at zero delay, i.e. the correlation coefficient, and the maximum of the cross-correlation function were recorded for each event. The allowed delay was chosen to maximize the separability between the spindle and control segments, computed as the proportion of correctly classified segments by a threshold in the correlation values. The threshold was varied to find an optimum value for each maximum delay.

The maximum of the normalized cross-correlation function depends on the shape of the envelope of the signals, but there could be a phase synchrony between the signals independently of their envelope. Therefore, the synchrony was also studied by analyzing the distribution of the local minima in the signals in an interval of ± 50 ms around the spindle onset. This onset was marked at a negative peak of the scalp signal. Then, channels in which a local minimum (negative peak) of the sigma band component is consistently found at a given delay with respect to the spindle onset will show a peak in the distribution of the delays. If the delay is not consistent among the different spindles, the distribution of the delays will be uniform, with no clear peak. We compare the distribution of the local minima in each channel to a uniform distribution with a non-parametric Kolmogorov–Smirnov goodness-of-fit hypothesis test, with a 5% significance level.

The same analysis was carried out for the spindles marked on intracranial channels in the hippocampus and insula.

The temporal coupling between neocortical spindles and hippocampal ripples has been hypothesized to serve a hippocampo-neocortical dialogue underlying memory consolidation during sleep (Siapas and Wilson, 1998; Clemens et al., 2007, 2011). To study the connection between scalp spindles and physiological ripples in the hippocampus, we looked for differences in the ripple band (80–250 Hz) energy in hippocampal channels during scalp spindles. The ripple band component of hippocampal channels was obtained with a zero-phase band-pass FIR filter (80–250 Hz, transition bands of width 10 Hz, 60 dB attenuation in the low frequency stop-band and 40 dB attenuation in the high frequency stop-band). Then, a paired t-test comparing the ripple band energy during scalp spindle and control intervals was performed. The same analysis was repeated including only fast scalp spindles with a frequency higher than 13 Hz, as fast centroparietal spindles are thought to be related to memory formation in contrast to more unspecific slow frontal spindles (Schabus et al., 2007). This analysis was repeated for hippocampal spindles.

## Results

### Distribution of evaluated intracranial EEG channels

In the 35 patients, we retained 280 intracranial EEG channels following the exclusion criteria indicated above. A median of 50 spindles (range, 31–50) in the scalp was analyzed. The median spindle rate was 4.0/min (range, 0.6–10). The frontal, parietal and temporal lobes were well sampled with ≥ 10 EEG channels per region. For detailed information on the sampled anatomical localizations see Fig. 1, Table 1, and Supplementary Fig. 1.

### Intracranial sigma energy at time of scalp spindles

Table 1 provides the percentages of channels with significant increase in sigma band energy at the time of scalp spindles compared to control intervals over the investigated brain regions. For this dataset, the Holm–Bonferroni correction for the 280 channel comparisons indicated an uncorrected p-value of 2.7 × 10^− 4^ for 5% significance. Sigma energy increased not only in the frontal and parietal neocortical lateral convexities, but also in remote areas such as the mesial frontal and parietal structures. The percentage of channels with a significant increase in sigma energy ranged between 25.0 and 80.8%. The sigma energy difference was between 1.6 and 4.6 dB. In contrast in the temporal neocortex only a low percentage of channels with a significant sigma energy increase was found (maximum 12.5%, maximum sigma energy difference of 0.8 dB), and there was no significant increase in the temporal limbic structures. The percentage of significant increase in sigma band energy in the insula channels showed an intermediate level of 25.0% with a sigma energy difference of 2.0 dB. We found no significant difference (unpaired t-test, p = 0.56) in the average sigma energy increase between the intracranial channels in the left (161 channels) and right (119 channels) hemispheres. A grouping of the scalp spindles in fronto-central, centro-parietal, and widespread spindles was performed (widespread if increase in sigma energy during the spindle was at least 6 dB in both regions, otherwise the region is determined by the largest increase in sigma energy when comparing fronto-central and centro-parietal channels). The mean intracranial sigma energy increase for the 570 (33.7%) widespread spindles was 3.6 dB, 1.3 dB for the 773 (45.7%) fronto-central spindles, and 1.1 dB for the 348 (20.6%) centro-parietal spindles.

The frequencies of peak sigma energy in the intracranial EEG at the time of scalp spindles followed a caudo-rostral gradient, with faster frequencies in the centro-parietal regions and slower frequencies in the frontal regions (Table 1).

**Fig. 2:**
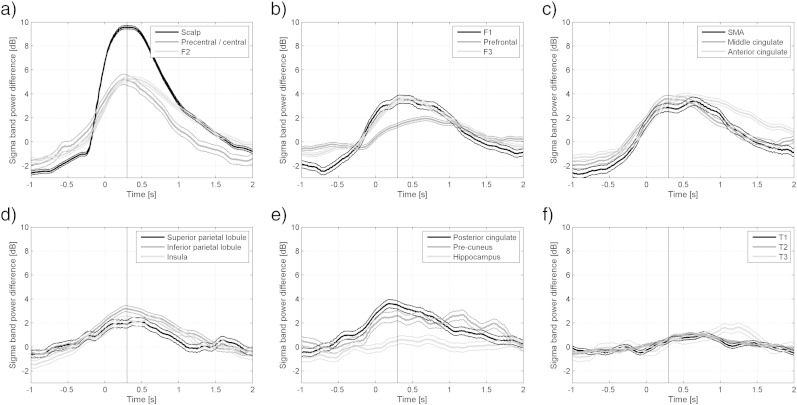
Evolution of sigma band energy in different brain regions during scalp spindles compared to control intervals. The evolution of all channels in each brain region was averaged. Time zero corresponds to the marked onset of the scalp spindles. The vertical line at time 0.30 s corresponds to the maximum average energy increase on the marked spindles. (a) + (b) Scalp and fronto-lateral regions. (c) Fronto-mesial regions. (d) Parieto-lateral regions and insula. (e) Parieto- and temporo-mesial regions. (f) Temporo-lateral regions.

Fig. 2 shows the changes over time of the sigma energy compared to the control intervals. Time zero corresponds to the onset of the spindles in the scalp. The maximum energy in the marked spindles was on average 9.5 dB (9 times) higher than in the control intervals, and occurred 0.30 s after onset. As expected, the brain regions with higher percentages of channels showing significant increases in sigma energy in Table 1 also expressed higher energy in Fig. 2. The energy in most of the regions peaked at approximately the same time as the scalp energy, except that the prefrontal region (Fig. 2b) and the anterior cingulate gyrus (Fig. 2c) showed later peaks.

**Fig. 3:**
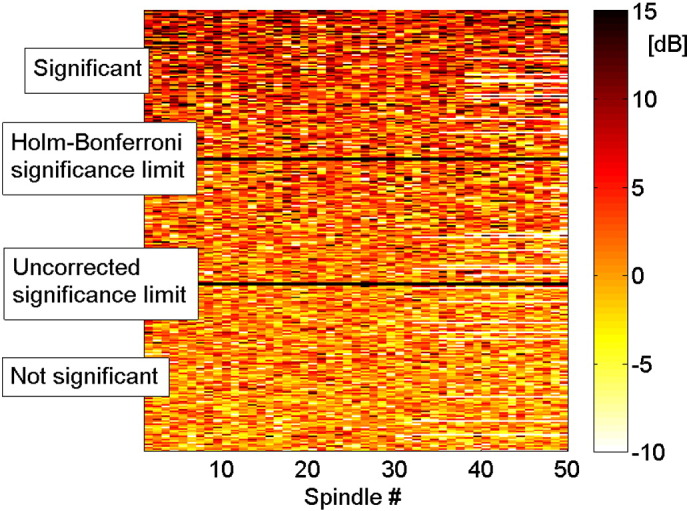
Sigma band energy variation (in dB) during scalp spindle intervals compared to control intervals for individual spindles. Each row corresponds to a different intracranial channel and each column to a single scalp spindle. The channels are sorted according to the p-value in a paired t-test comparing the sigma band energy in the spindle and control intervals. The white spaces correspond to channels of subjects with fewer than 50 marked scalp spindles. Channels show a large variability in sigma band energy increase at the time of different scalp spindles. More significant channels are more likely to exhibit an increase in the sigma band energy, but such increases are also seen occasionally on non-significant channels.

To determine if the described average behavior corresponds to a consistent pattern for individual spindles, in Fig. 3 we show the increase in sigma energy of every individual spindle for each of the 280 intracranial channels. Each row in the matrix depicted in Fig. 3 corresponds to a channel and each column to a scalp spindle. The color level indicates the change in sigma energy of each spindle interval of 1 s compared to the corresponding control intervals. These are the values used for the hypothesis test reported in Table 1, and the channels are sorted according to p-value. In any single channel there was a large variability among the different spindle intervals: channels with lower p-values correspond to a higher probability of sigma energy increase, but there is no clear stable pattern. In some intervals we found a decrease in sigma energy, which can be explained by the presence of spindles in intracranial channels during the spindle-free control intervals in the scalp EEG (4% of the total number of intervals showed a decrease of sigma band energy below − 6 dB, and these intervals had on average a 6 dB increase in the energy during the control interval, compared to the rest of the control intervals). We conclude that the spatial distribution of increases in sigma energy is highly variable from spindle to spindle.

**Fig. 4:**
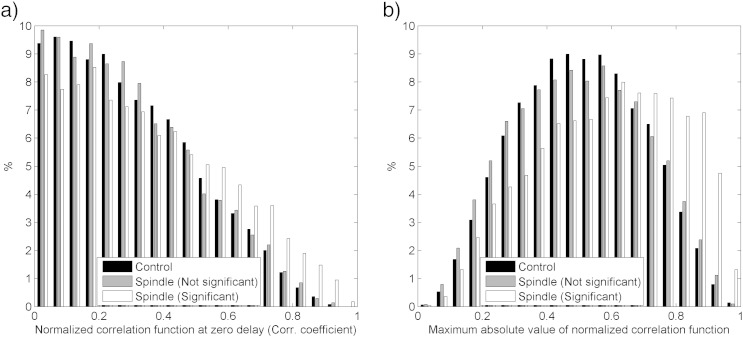
Proportion of scalp spindles with different degrees of cross correlation with the intracranial channels. The results are shown separately for control intervals, spindle intervals in channels showing no statistically significant increase of sigma band energy during scalp spindles, and spindle intervals in channels with a statistically significant increase of sigma band energy during scalp spindles. (a) Normalized cross-correlation function at zero delay (i.e. cross correlation coefficient). (b) Maximum of the absolute value of the normalized cross correlation (for delays between ± 28 ms). A higher correlation can be observed for channels with significant increase of sigma activity during scalp spindles, but a large proportion of them show a low correlation coefficient. The correlation increases if a delay is allowed between the scalp and intracranial channels, but this improvement is not specific to spindles, as it is also observed during the control non-spindle segments.

We also studied the degree of synchrony between the scalp spindles and the intracranial sigma activity. Fig. 4a shows a histogram of the correlation coefficient between scalp spindles and intracranial sigma activity. The proportion of cross-correlation coefficients in different range bins is shown for the control intervals and for the time of scalp spindles; the latter is divided into the intracranial channels not showing a significant increase in sigma energy at the time of scalp spindles (p > 0.05) and the ones showing a significant increase (p < 0.05). The cross-correlation coefficients for the control intervals and the non-significant channels had an almost equal distribution, while the channels with a significant energy increase had slightly higher correlation coefficients. However, the proportion of cases with high correlation coefficients was low in the channels with significant sigma power increases, the channels with no significant sigma power increases, and the control intervals, indicating a poor synchrony between intracranial sites and scalp EEG. If a delay was allowed between scalp and intracranial signals, the maximum values of the cross-correlations were higher as seen in Fig. 4b, but the differences between the significant channels and the control intervals or the non-significant channels were still low, even when choosing a maximum delay of 28 ms, which maximized the separability of the spindle and control segments.

**Fig. 5:**
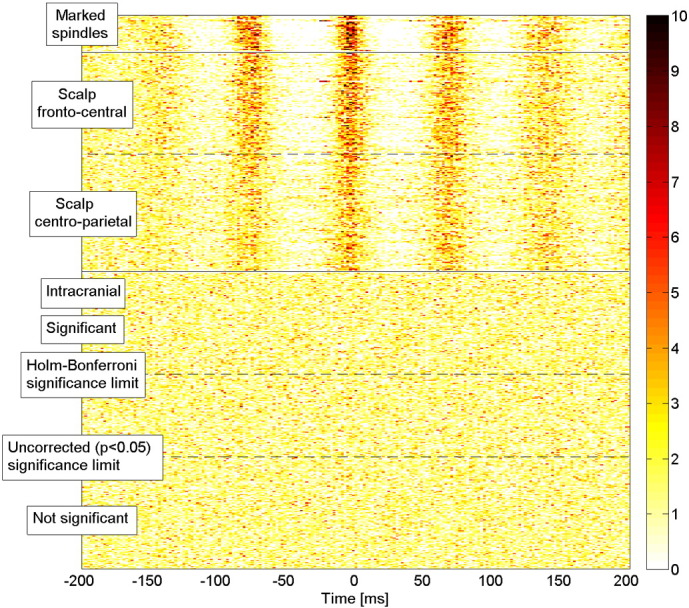
Histogram of local minima around scalp spindles. Each row corresponds to a channel and each column to a 2 ms bin. Time zero corresponds to the marked onset of the spindles. The color indicates the number of local minima in each bin, accumulated for all the spindle intervals (50 spindle intervals in most cases). The top 35 rows correspond to the marked spindles, the middle rows to other scalp channels, and the bottom half to the intracranial channels, sorted by p-value in the paired t-test comparing the sigma band energy in the spindle and control intervals. Synchrony is consistently observed in the scalp channels but not in the intracranial channels, indicating that for every spindle the delay between scalp and intracranial channels is different.

**Fig. 6:**
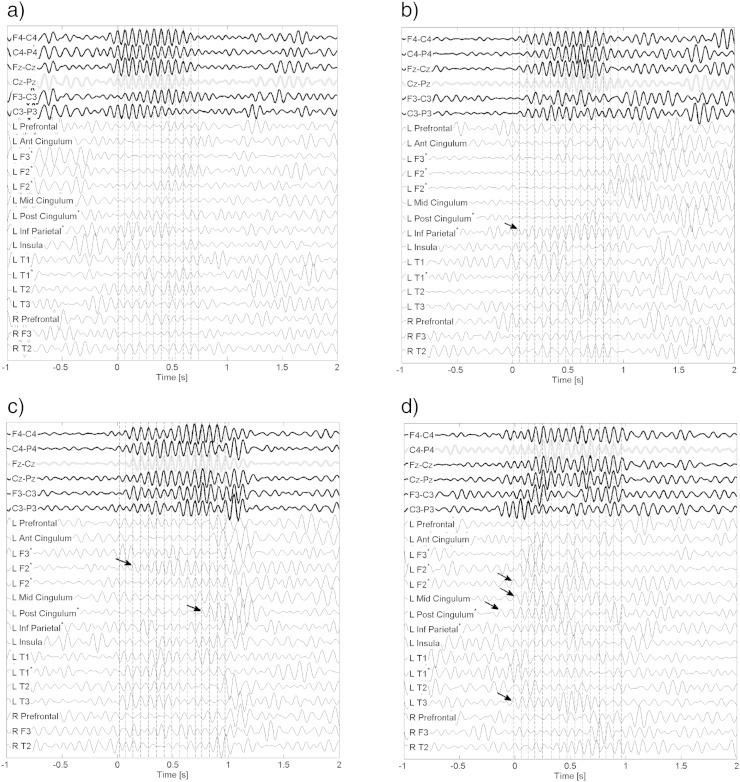
Examples from simultaneous scalp EEG spindles and intracranial spindle activity. Only the sigma band component (10–16 Hz) is shown. Four examples from spindles of the same subject (ID 29) are shown. The scalp channel with the gray trace is the channel in which the spindle was marked. Time zero is the marked spindle onset. Other scalp channels are shown in black, and intracranial channels are shown in gray. The brain regions from which the channels are recorded are indicated on the right, a star indicates that the channel showed significant increase in sigma band energy compared to the control intervals in the average of the 50 spindles. Arrows indicate intracranial sigma activity that fulfills the criteria to be considered a spindle. The examples demonstrate that even though in some channels there was a frequent increase of sigma band energy at the same time as in the scalp spindles, the involved channels and underlying synchrony are not consistent.

A high maximum absolute value of the normalized correlation function indicates that the signals in the intracranial and scalp channels are similar except for a delay. However, the cross-correlation results do not indicate if the delay is always the same for different spindles in any given intracranial channel. To determine if there is a consistent delay, we looked for local minima in the sigma band component of the signals. For each channel we counted the number of local minima in 2 ms intervals, accumulated for all spindle intervals. The results are shown in Fig. 5. Each row in the matrix corresponds to a channel and each column to a time bin relative to the marked spindle onset. The color level indicates the number of local minima in each bin. The results for the marked spindles for the 35 subjects are shown at the very top. The spindle onset was marked at the first clear negative peak of the signal using a filter with a pass band between 5 and 20 Hz, and as expected a very good synchrony is observed for the marked spindles. In most cases there is a local minimum one wave length before the marked onset, although not a clear one. As time passes, the synchrony decreases due to differences in spindle frequencies and occurrence of spurious local minima. In the middle section of the figure we see that the remaining scalp channels also show a high degree of synchrony with the marked spindles. The lower half corresponds to the 280 intracranial channels, again sorted according to the p-value in the sigma band energy test. No clear pattern is seen in these channels, indicating that there was no consistent delay for different spindles in any channel, and no clear similarities among channels. We performed a statistical test comparing the distribution of the minima in a ± 50 ms interval around the marked onsets to a uniform distribution. At a 5% significance level, we found evidence for rejecting the hypothesis of uniformly distributed minima in 33 of the 35 (94%) on the marked spindle sequences. In the 206 scalp channels, the distribution of the minima was significantly different from a random distribution in 151 (73%) of the cases, but only in 10 of the 280 intracranial channels (3.6%). In 6 of these 10 channels there was also significant increase in sigma band energy in the spindle intervals (4 neocortical, 2 in the cingulate gyrus). This means that even though in some channels there was a frequent increase of sigma band energy at the same time as the scalp spindles, the channels showing this increase and underlying synchrony were not consistent. This is illustrated by four typical examples of individual scalp spindles of one patient in Fig. 6. We conclude that there is very low synchrony between the scalp spindle and the intracranial events, even if one introduces a delay; in addition, the synchrony is highly variable from event to event.

To study the connection between scalp spindles and physiological ripples in the hippocampus, we looked for differences in the ripple band (80–250 Hz) energy in hippocampal channels during scalp spindles and the corresponding control non-spindle segments. The t-test comparing ripple band energy in the hippocampal channels revealed no evidence to reject the null hypothesis of equal energy (p = 0.09). The same lack of evidence (p = 0.14) was obtained when only taking into account the fast scalp spindles (spindles with a frequency > 13 Hz).

In summary, we found an increase of the sigma activity in intracranial channels during scalp spindles. On average this increase was more likely in some brain regions, but the involved channels varied in each spindle. Also, even if the scalp spindle and intracranial events were simultaneous, the phase synchronization of the waves was very poor, and highly variable in each spindle.

### Scalp and intracranial sigma energy at the time of hippocampal and insula spindles

In this part of the project, we investigated the subgroups of the 35 patients who had normal hippocampal (n = 7) or insula (n = 8) intracranial EEG channels.

#### Hippocampal spindles & sigma energy in scalp and intracranial EEG

At least 5 unambiguous spindles were found in six of the 7 patients without abnormal hippocampal EEG activity (median, 31.5; range, 7–50). The median hippocampal spindle rate was 0.6/min (range, 0.1–6.7). A total of 70 intracranial EEG channels and 38 scalp EEG channels were investigated for simultaneous sigma energy increase at time of hippocampal spindles. The regions sampled with ≥ 5 channels were the frontal lobe [prefrontal area, frontal neocortex (first, second and third frontal gyri), and anterior and midcingulate gyrus], the temporal lobe (temporal neocortex) and scalp (fronto-central, centro-parietal). For this dataset an uncorrected p-value of 5.8 × 10^− 4^ corresponded to 5% significance according to the Holm–Bonferroni correction for the 108 comparisons.

Table 2 shows the percentages of a simultaneous increase in sigma energy in the non-hippocampal intracranial and scalp channels. At the time of hippocampal spindles, 66.7% of temporo-mesial structures showed an increase in sigma energy with a difference of 3 dB between spindle and control segments. Further regions with moderate levels of sigma energy increase between 25.0 and 42.9% were the temporal neocortex, the frontolateral neocortex and precentral/central regions, followed by the scalp between 18.8 and 22.7%. For all other extratemporal channels the percentages of channels with an increase in sigma energy at time of hippocampal spindles were comparatively low (Table 2).

**Fig. 7:**
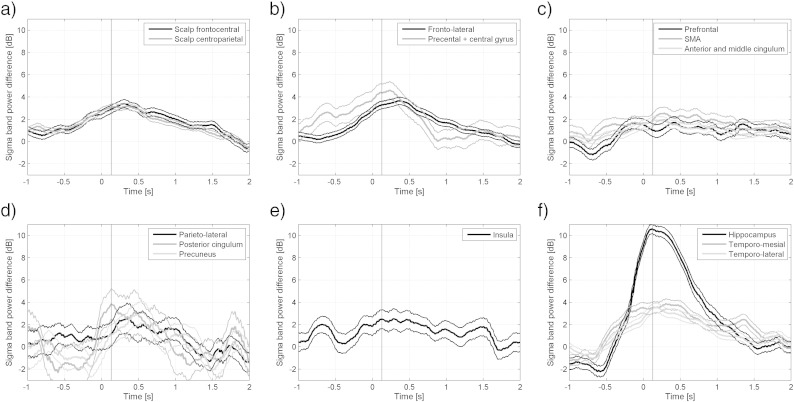
Evolution of sigma band energy in different brain regions during hippocampus spindles compared to control intervals. The evolution of all channels in each brain region was averaged. Time zero corresponds to the marked onset of the hippocampus spindles. The vertical line at time 0.13 s corresponds to the maximum average energy increase of the marked spindles. (a) Scalp. (b) Fronto-lateral regions. (c) Fronto-mesial regions. (d) Parietal regions. (e) Insula. (f) Temporal regions.

The time evolution of the average energy in the sigma band is shown in Fig. 7. The maximum of the increase in sigma energy compared to control intervals was similar to that in the scalp spindles, 10.5 dB or 11 times, but the hippocampal spindles were on average of much shorter duration. The peak in sigma power occurred 130 ms after onset, and the energy decreased rapidly. Given the lower number of channels, the energy signals had more noise and usually showed several peaks, but no brain region showed a change earlier than the hippocampus, and the energy increase in the parietal regions seemed delayed with respect to the hippocampus. No synchrony was observed between hippocampal spindles and sigma energy in other channels. A significant difference from a random distribution of the minima around the marked onset was found only in one of the 38 (2.6%) scalp channels, and in two of the 64 (3%) intracranial channels, but these channels did not show a significant increase in sigma energy during the spindle intervals, suggesting that they may be false positives (compatible with the 5% significance level).

To study the connection between hippocampal spindles and physiological ripples in the hippocampus, we looked for differences in the ripple band (80–250 Hz) energy in hippocampal channels during hippocampal spindles and the corresponding control non-spindle segments. The t-test comparing ripple band energy in the hippocampal channels revealed no evidence to reject the null hypothesis of equal energy (p = 0.11). However, an increase of ripple band energy was observed in 4 out of 6 patients. This increase was not enough to reach statistical significance given the low statistical power associated with the limited number of healthy hippocampal channels in our dataset.

#### Insula spindles & sigma energy in scalp and intracranial EEG

At least 5 unambiguous spindles were found in seven of the 8 patients without abnormal insula EEG activity (median, 50; range, 26–50). The median insula spindle rate was 0.8/min (range, 0.5–4.5). A total of 82 intracranial EEG channels and 42 scalp EEG channels were investigated for a simultaneous increase in sigma energy at time of insula spindles. The regions sampled with ≥ 5 channels were located in the frontal lobe [frontal neocortex (first, second and third frontal gyri), precentral/central region, anterior and midcingulate gyrus and supplementary motor area], parietal lobe (superior and inferior parietal lobules), temporal lobe (temporal neocortex and temporo-mesial limbic structures) and scalp (fronto-central, centro-parietal). Table 3 shows the percentages of a simultaneous increase in sigma energy in the non-insula intracranial and scalp EEG channels. For this dataset an uncorrected p-value of 7.1 × 10^− 4^ corresponded to 5% significance according to the Holm–Bonferroni correction for the 124 comparisons.

The highest percentages of channels with significant increase in sigma energy between 60.0 and 66.7% were found in the frontal lobe (maximum sigma energy difference 4.7 dB) followed by the scalp (maximum 52.2%, maximum sigma energy difference 3.0 dB) and the parietal lobules (maximum 33.3%, maximum sigma energy difference 2.0 dB). Increases in sigma energy in the temporal lobe were scarce with a maximum of 20% in the temporo-lateral structures (sigma energy difference 1.0 dB).

**Fig. 8:**
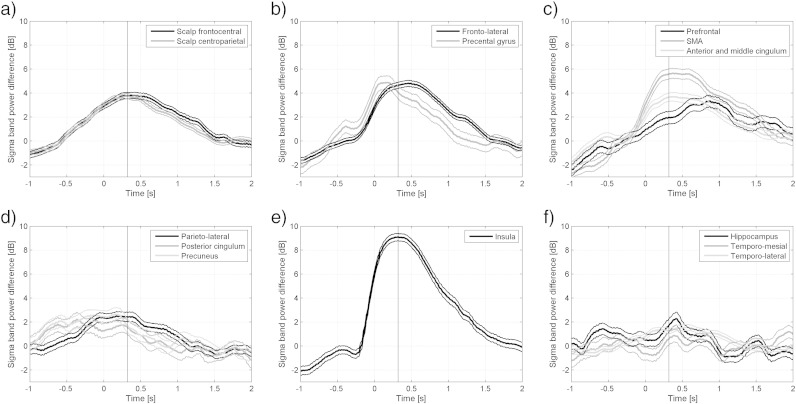
Evolution of sigma band energy in different brain regions during insula spindles compared to control intervals. The evolution of all channels in each brain region was averaged. Time zero corresponds to the marked onset of the insula spindles. The vertical line at time 0.32 s corresponds to the maximum average energy increase of the marked spindles. (a) Scalp. (b) Fronto-lateral regions. (c) Fronto-mesial regions. (d) Parietal regions. (e) Insula. (f) Temporal regions.

We show the time evolution of the sigma energy in Fig. 8. The insula spindles were of similar duration as the scalp spindles, with the peak in energy 320 ms after onset, with a similar average energy increase compared to the control intervals, around 9.0 dB (8 times). The number of channels was comparatively low and the resulting signals were noisy, but the impression is that the increase in sigma energy occurs slightly earlier in the parietal lobe channels, and slightly later in the prefrontal region. The highest response in the supplementary motor area peaks at approximately the same time.

Only one of 42 (2.4%) scalp channels showed evidence of a non-uniform distribution of the minima around the marked insula spindle onset. More evidence of synchrony was found between insula spindles and other intracranial channels, with evidence of non-uniform distribution of the minima in 12 of 64 channels, all of them also showing a significant increase of sigma energy. Eight of these channels were neocortical (6 frontal, 1 parietal, 1 temporal), and four were mesial frontal (2 in the supplementary motor area, 1 prefrontal, and 1 in the cingulate gyrus). This suggests some degree of synchrony between the insula and other brain structures, although the statistical test only hints to some degree of consistency in the delay between the spindle onset and the closest minima in other channels. This does not imply that the actual delay and spindle frequencies are the same between channels.

In summary, as in the case of scalp spindles, the most striking characteristic of the correlates of hippocampal and insula spindles is its lack of consistency among the individual events, showing variable involvement and low synchrony of channels in other brain regions.

## Discussion

This is the first study to directly investigate intracranial correlates of spindles in the surface EEG with combined scalp and intracranial EEG, and the scalp correlates of spindles in the hippocampus and the insula as examples of brain structures remote from the scalp. Major findings are that spindles in the scalp are accompanied by widespread cortical increases in sigma band energy (10–16 Hz) with highest percentages of this increase in the frontoparietal lateral and mesial neocortex. The intracranial involvement during scalp spindles showed no consistent pattern, and exhibited unexpectedly low synchrony across brain regions. In contrast to scalp spindles, hippocampal spindles were shorter and more spatially restricted with low synchrony, even within the temporal lobe. Similar results were found for the insula.

### Widespread increase in intracranial sigma energy with scalp spindles

We found a widespread increase in intracranial sigma band energy at the time of scalp spindles. This finding was not only evident over the fronto-centro-parietal lateral neocortex, but also in more remote brain areas such as the orbitofrontal region, the supplementary motor area, the cingulate gyrus, and the pre-cuneus region. This widespread distribution of energy in the sigma band at time of scalp spindles is best explained by the widespread extent of thalamo-cortical projections (Steriade, 2006). In contrast, the neocortical and mesiolimbic temporal structures showed low or no significant increase in sigma band energy at the time of scalp spindles. This finding might follow the notion that connections between the dorsal thalamic nuclei which project to the mesiotemporal structures are not reciprocally connected with the reticular nucleus responsible for spindle generation, in contrast to thalamic nuclei projecting to the frontal and parietal lobe structures. In addition, the amygdala is sparsely connected to the thalamus (Jones, 2007). A low increase of sigma activity in the temporal neocortex is more difficult to explain, as at least in the cat and rat, it is well connected with the pulvinar nucleus which is reciprocally connected with the reticular nucleus (Jones, 2007).

Several studies in humans demonstrated that spindles are widely spread over different cortical areas with the lowest spindle rates seen in the mesiolimbic temporal structures (Nakabayashi et al., 2001; Nakamura et al., 2003; Andrillon et al., 2011; Peter-Derex et al., 2012). Two groups found no neocortical temporal spindles (Nakabayashi et al., 2001; Peter-Derex et al., 2012) but one group showed similar spindle rates in the temporal as in the frontal and parietal neocortex (Andrillon et al., 2011). Following a different approach, the present study also demonstrated sigma activity increase in the temporal neocortex, but only in a maximum of 12.5% of channels. Although epochs with interictal epileptic discharges were discarded in the previous studies, the evaluation of epileptic channels and the presence of a lesion may explain the difference observed in the results. In the present study, we excluded all channels which had more than very rare epileptiform activity, non-epileptiform anomalies, or which were located in brain lesions.

The peak of intracranial sigma energy was usually at the time of the peak of scalp spindles, except in the anterior cingulate gyrus and prefrontal region where it was delayed. This is in line with previous works (Andrillon et al., 2011; Peter-Derex et al., 2012) showing that centroparietal spindles occurred on average 200 ms earlier than frontal spindles. In addition, we confirmed the presence of a caudo-rostral gradient of intracranial sigma energy at the time of scalp spindles: sigma frequencies in frontal structures are lower than sigma frequencies in centroparietal structures. This result also confirms that of previous authors who investigated spindle rates and frequencies over different intracranial regions (Andrillon et al., 2011; Peter-Derex et al., 2012), and is in line with scalp quantitative EEG studies demonstrating fast parietal spindles and slow frontal spindles (Anderer et al., 2001).

### Do spindles have a consistent intracranial correlate?

We found that individual sleep spindles are highly variable concerning the increases in sigma band energy in intracranial structures. One explanation comes from the recent concept of human sleep as a local phenomenon (Krueger et al., 2008): the reticular nucleus in the thalamus generates sleep spindles (Fuentealba and Steriade, 2005), but the expression of the thalamic input is modulated by the activation state of the individual cortical columns. This hypothesis is in line with the studies of Steriade (2006) demonstrating in the cat model that sleep spindles occur predominantly during the “up” or depolarizing state of the slow waves. Moreover, intracranial spindles were recently shown to be more local than generalized during human sleep (Andrillon et al., 2011).

### Synchrony of spindles

We studied synchrony of spindles by calculating the cross-correlation function and the distribution of the local minima in the signals in an interval of ± 50 ms around the spindle onset. Interestingly, we found a low synchrony of intracranial sigma activity in contrast to synchronous spindles in the scalp EEG channels. Multiple asynchronous spindles were described with MEG, which is in contrast to scalp EEG findings (Dehghani et al., 2011). Moreover, during cat experiments, cortical barbiturate spindles were shown to appear either as local spindles in restricted cortical areas or as generalized spindles over several areas. However, even generalized spindles were clearly different with regard to intra-spindle wave frequency, duration, and the time of start and stop of the spindles among different lobes, and more pronounced between both hemispheres (Andersen et al., 1967).

A local cortical influence on spindle generation presents one potential explanation for the low synchrony of intracranial spindles (Krueger et al., 2008). The role of laminar-dependent cortico-thalamic projections or thalamo-cortical projections via the core or matrix cells of the dorsal thalamus could represent alternative or additional explanations to our findings (Jones, 2009). Also, the presence of relatively simultaneous but not synchronized spindles generated in many brain regions may result in a scalp spindle, due to the summation effect of the skull. In line with this a recent study in monkeys demonstrated that the scalp EEG power was explained by both synchrony (spectral coherence) and amplitude of the intracranial activity, with synchrony contributing 40% and amplitude 60% (Musall et al., 2014). Hence, the observed widespread increase in amplitude of asynchronous sigma band activity could be sufficient to explain the presence of synchronous scalp spindles. Further studies should be pursued to confirm this hypothesized mechanism of scalp spindle generation.

### Association of scalp spindles, hippocampal spindles & hippocampal ripple band energy

The role of fast centroparietal spindles for memory consolidation and learning has been demonstrated by different groups (Moelle and Born, 2011; Fogel and Smith, 2011; Ferrara et al., 2012). A temporal coupling between slow oscillations, spindles and ripples may serve hippocampo-neocortical communication resulting in memory consolidation during sleep. This temporal coupling was only recently demonstrated in humans who underwent EEG recording with foramen ovale electrodes in the setting of preoperative epilepsy evaluations (Clemens et al., 2007, 2011). The authors found that ripple activity was increased before spindle peaks and distinctly decreased after the peak. We investigated a different perspective by comparing the amount of ripple band activity during scalp spindles as well as hippocampal spindles, and ripple band activity during non-spindle control segments. We found no significant difference in the hippocampal ripple band energy at time of scalp or hippocampal spindles and control intervals. This makes it unlikely that the relationship between spindles and limbic function is mediated by spindle-facilitated ripples. It does not exclude, however, another form of functional relationship between spindles and limbic function for memory consolidation during sleep.

### Scalp and intracranial sigma energy at the time of hippocampal or insula spindles

In contrast to the usually widespread increase in sigma activity at the time of scalp spindles, no simultaneous sigma energy increase was observed in the mesiolimbic structures. This finding can either be explained by a general lack of spindles in the hippocampus, or hippocampal spindles that are not time-locked to the scalp spindles. We found that hippocampal spindles are largely independent of scalp spindles. They are shorter and less frequent. Since we only investigated channels of normal hippocampi (as based on patients' history, MRI results, and interictal and ictal findings), we are confident that these spindles are a physiological and not an epileptic phenomenon (Montplaisir et al., 1981; Malow et al., 1999). The issue of whether hippocampal spindles are also present in the epileptic hippocampus awaits further investigation, and cannot be answered by the present data.

Hippocampal spindles were more spatially restricted compared to a widespread sigma energy distribution at the time of scalp spindles. Increases in sigma band energy with hippocampal spindles were mostly found within the temporal lobe and to a small extent in the frontal areas and the scalp. In line with our findings, Sarasso et al. (2014) demonstrated that hippocampal spindles are already present minutes before the first spindle seen in the scalp. The fact that we did not find a simultaneous increase of sigma band energy in the parietal lobe should be interpreted with caution, as data derive from only seven parietal intracranial bipolar channels.

Spindles in the insula were investigated to assess another remote brain structure. We found that spindles were present in the insula with spindle rates between those of the hippocampus and the scalp EEG. At time of insula spindles a widespread increase in sigma energy was found in all evaluated areas with lowest increases in the temporal lobe.

### Limitations

Intracranial EEG recording in the setting of pre-surgical epilepsy evaluation is a unique opportunity to study directly intracerebral neuronal activity, acknowledging that these recordings are limited to epilepsy patients with all or part of their usual antiepileptic medication. To partially overcome the problem of studying potentially abnormal brain, two electrophysiologists selected only channels with no or rare epilepsy potentials as well as no non-epileptic anomalies, and located in normal looking MRI regions. Also, the fact that scalp spindle rates corresponded to that of healthy persons (Nicolas et al., 2001) makes us confident that our findings reflect normal sleep physiology. Moreover, due to the special setting of invasive EEG recording, scalp spindles were detected in a bipolar montage along the antero-posterior axis, as done by other authors working with depth electrode recordings (e.g. Sarasso et al., 2014), and not in a referential montage to the mastoid as recommended for sleep recordings. Even if a mastoid electrode was available, it could cause problems in temporal lobe epilepsy as the mastoid records epileptic activity from the temporal lobe. To account for the reference issue, we checked prior to this study whether conventional sleep recordings with the same electrodes using a bipolar and mastoid reference montage would reveal the same spindle events. In line with Werth et al. (1997) we found that most spindles seen in the bipolar montage can be also seen in a mastoid referential montage. The study may have suffered from a poor resolution due to limited sampling. We have adequately sampled the frontal, parietal and temporal neocortices, but less adequately the mesial temporal structures, the insula and the occipital cortex. However, the present study reports data on 35 patients which represents the largest group reported for intracranial sleep analysis. Another limitation is that the scalp EEG was limited to nine electrodes placed at frontal, central and parietal positions.

## Conclusions

This study demonstrated widespread intracranial sigma activity at the time of spindles in the scalp. This intracranial sigma activity showed a high variability across individual spindles, and a low synchrony compared to the synchronized spindles seen in scalp EEG. Hippocampal spindles were, on the other hand, more locally restricted. Our findings suggest that the generation of spindles is under a higher than expected focal cortical influence contributing to the concept of sleep as a local phenomenon and challenging the notion of spindles as a widespread synchronous discharge.

## Funding

This work was supported by the Austrian Sleep Research Association, the Austrian Science Fund (Schrödinger fellowship abroad J3485 to BF), and the Canadian Institutes of Health Research (grant MOP-10189).

## Figures and Tables

**Table 1:** Spindles identified in the scalp & simultaneous occurrence of sigma band energy increase over the investigated brain regions.

Region	Number of channels analyzed	Significant channels corrected per channel	Significant channels corrected per region	Significant channels uncorrected	Sigma power diff [dB]	Frequency mean [Hz]	Frequency SD [Hz]
Prefrontal	22	27.3	54.5	63.6	1.6	12.0	0.6
F1	9	33.3	88.9	88.9	3.1	12.5	1.1
F2	26	80.8	96.2	96.2	4.6	12.4	0.8
F3	31	48.4	64.5	87.1	3.1	12.2	0.9
Precentral/central	7	57.1	100.0	100.0	3.8	13.5	1.2
Anterior cingulate	18	55.6	77.8	77.8	3.3	11.6	0.8
Middle cingulate	11	45.5	81.8	81.8	3.0	13.0	0.9
SMA	9	55.6	66.7	77.8	2.8	12.2	1.6
Superior parietal	8	25.0	37.5	62.5	1.8	12.5	1.1
Inferior parietal	12	50.0	83.3	91.7	2.7	12.5	1.4
Pre-cuneus	4	75.0	75.0	75.0	2.3	13.3	1.3
Posterior cingulate	8	37.5	87.5	87.5	3.0	13.9	0.8
Insula	8	25.0	37.5	50.0	2.0	12.9	1.4
T1	16	6.3	12.5	25.0	0.6	12.5	1.5
T2	72	12.5	12.5	38.9	0.8	11.2	1.3
T3	5	0.0	0.0	0.0	0.5	NA	NA
Heschl gyrus	1	0.0	0.0	0.0	0.7	NA	NA
Hippocampus	7	0.0	0.0	0.0	0.4	NA	NA
Amygdala	2	0.0	0.0	0.0	0.1	NA	NA
Entorhinal cortex	1	0.0	0.0	0.0	− 0.4	NA	NA
Fusiform gyrus	2	0.0	0.0	0.0	0.5	NA	NA
Cuneus	1	0.0	0.0	0.0	0.6	NA	NA

**Table 2:** Spindles identified in the hippocampus & simultaneous sigma band energy increase over other brain regions and the scalp.

Region	Number of channels analyzed	Significant channels corrected per channel	Significant channels corrected per region	Significant channels uncorrected	Sigma power diff [dB]	Frequency mean [Hz]	Frequency SD [Hz]
Prefrontal	6	0.0	16.7	33.3	1.4	12.0	0.3
Fronto-lateral	19	26.3	36.8	52.6	2.7	12.7	1.5
Precental + central gyrus	4	25.0	25.0	75.0	2.6	12.6	2.3
Anterior + middle cingulate	12	8.3	25.0	33.3	1.7	11.3	1.0
SMA	3	0.0	66.7	66.7	2.2	13.3	3.0
Parieto-lateral	3	0.0	0.0	0.0	2.0	NA	NA
Pre-cuneus	3	0.0	0.0	0.0	1.7	NA	NA
Posterior cingulate	1	0.0	0.0	0.0	2.0	NA	NA
Insula	3	0.0	33.3	33.3	1.5	13.2	0.0
Temporo-lateral	7	42.9	57.1	71.4	2.7	11.4	1.9
Temporo-mesial	3	66.7	100.0	100.0	3.0	11.6	2.3
Scalp frontocentral	16	18.8	25.0	50.0	2.4	12.4	1.4
Scalp centroparietal	22	22.7	36.4	50.0	1.9	12.2	1.4

**Table 3:** Spindles identified in the insula & simultaneous occurrence of sigma activity over other brain regions and the scalp.

Region	N of channels analyzed	Significant channels corrected per channel	Significant channels corrected per region	Significant channels uncorrected	Sigma power diff [dB]	Frequency mean [Hz]	Frequency SD [Hz]
Prefrontal	4	25.0	75.0	75.0	2.2	11.1	0.6
Fronto-lateral	21	61.9	71.4	71.4	3.5	12.2	1.2
Precental gyrus	5	60.0	60.0	80.0	3.2	12.8	2.8
Anterior + middle cingulate	8	62.5	75.0	75.0	3.3	11.9	1.1
SMA	6	66.7	100.0	100.0	4.7	12.4	1.5
Parieto-lateral	6	33.3	33.3	66.7	2.0	12.2	1.6
Pre-cuneus	3	33.3	33.3	33.3	1.4	11.6	0.0
Posterior cingulate	2	0.0	50.0	50.0	0.8	10.2	0.0
Temporo-lateral	15	20.0	26.7	33.3	1.0	11.2	1.4
Temporo-mesial	2	0.0	0.0	0.0	0.1	NA	NA
Scalp frontocentral	19	36.8	57.9	73.7	3.0	12.2	0.9
Scalp centroparietal	23	52.2	56.5	65.2	2.8	12.3	1.0

## Appendix A Supplementary data

Supplementary material The Supplementary material comprises clinical information on the investigated patient sample (Table 1) and the localization of the electrode contacts and their sigma band power increases during scalp spindles for each patient (Figure 1).
