# Supplementary material for: Scalp spindles are associated with widespread intracranial activity with unexpectedly low synchrony
Source: Neuroimage. 2015 Jan 15;105:1–12. doi: 10.1016/j.neuroimage.2014.10.048 (PMC4275575; doi:10.1016/j.neuroimage.2014.10.048)
Supplement: Supplementary material — The Supplementary material comprises clinical information on the investigated patient sample (Table 1) and the localization of the electrode contacts and their sigma band power increases during scalp spindles for each patient (Figure 1). [file mmc1.docx]

**ONLINE SUPPLEMENTARY MATERIAL**

**Table 1.** Clinical characteristics of the patient sample investigated in this study

|  | **Sex** | **Age** | **Scalp EEG** | **SOZ SEEG** | **MR imaging** | **Antiepileptic medication**  **(mg / day)** | **Surgery (area resected)** | **Spindle rate** | **Analyzed spindles** |
| --- | --- | --- | --- | --- | --- | --- | --- | --- | --- |
|  |  |  |  |  |  |  |  |  |  |
| 1 | m | 47 | no IEDs | L F2 | Remote L F1 and L ant T resections | TPM (400), OXC (3000) | L F1 and F2 | 7.7 | 50 |
| 2 | m | 38 | R FT | R F3 | FCD R F3 | CBZ (1000), CLO (30) | R lateral F 3 | 3.6 | 50 |
| 3 | m | 21 | R T | R T mesial | R MTS; L inf O pole gliosis | NA | R ant T and AH | 3.8 | 50 |
| 4 | f | 24 | R T | R T mesial and neocortical | L F1 hyperintensity | CBZ (1200) | R SeAH | 1.2 | 50 |
| 5 | m | 41 | R FCT | R SMA and central | no lesion | OXC (1200), TPM (400), Clon (0.5) | no | 4.6 | 50 |
| 6 | f | 41 | L T | L T mesial | no lesion | CBZ (1200), LTG (400) | L SeAH | 3.1 | 50 |
| 7 | f | 40 | bil T | R mesial T; L Fus and T neocortex | bilateral focal nodular heterotopia: R F, R and L atrium, L O horn with TO abnormal gyration | CLO (25), LEV (3000) | no | 2.7 | 50 |
| 8 | f | 29 | R FT | R OF, FT and T | FCD R OF | NA | R OF | 3.6 | 50 |
| 9 | m | 30 | L CPT | L post Ins and post perisylvian neocortex | L post perisylvian atrophy and gliosis | LTG (600), LEV (1500), TOP (400) | L inf P | 10 | 50 |
| 10 | f | 20 | L P | L precuneus | FCD L precuneus | CBZ (800), CLO (20), LTG (200) | L precuneus | 10 | 50 |
| 11 | f | 27 | R hemis-pheric, T max | R ant perisylvian and T neocortex | no lesion | LTG (400), LEV (1500) | R ant T and AH | 7.9 | 50 |
| 12 | m | 46 | L FT | L ant Ins and ant T neocortex | no lesion | CBZ (1200) | no | 4.2 | 50 |
| 13 | m | 35 | L T | L T + L perisplenial area > L Ins >>> L T mesial | no lesion | CLO (30), CBZ (600), TPM (200) | L ant T and A | 7.1 | 50 |
| 14 | f | 57 | R FT | R Ins | remote R T resection | LTG (200), CBZ (400), CLO (25) | R Ins and extended T removal | 1.9 | 50 |
| 15 | m | 34 | bil T | bil T neocortical and mesial | no lesion | CLO (40), TPM (200), CBZ (1800) | L ant T and AH | 6.3 | 50 |
|  |  |  |  |  |  |  |  |  |  |
| 16 | f | 38 | L T | L T neocortex and mesial | L H malrotation; L F encephalocele | LEV (2000), LAC (300) | L ant T and AH | 4.2 | 50 |
| 17 | m | 45 | bil T | bil T mesial, R>L | bil H atrophy; R F encephalomalacia | LTG (375), LEV (3000), CBZ (800), Clon (2.5) | R SeAH | 0.7 | 31 |
|  |  |  |  |  |  |  |  |  |  |
| 18 | m | 27 | bil T | R T mesial | no lesion | CBZ (1400), CLO (10) | R SeAH | 7.1 | 50 |
|  |  |  |  |  |  |  |  |  |  |
| 19 | m | 36 | L FT | L Ca and OF | FCD L ant cingulate | LEV 3000, CBZ 1600 | L ant cingulate | 2 | 50 |
| 20 | f | 39 | R T | R T neocortical and mesial | R T atrophy | CBZ (1200), CLO (30) | R SeAH | 4 | 50 |
| 21 | f | 28 | L F | L F2 | FCD L F2 | CBZ (1600), CLO (10) | L F2 | 3.6 | 50 |
| 22 | m | 26 | R FT | R F3 | FCD R F3 | LEV (3000), CBZ (1200) | R F3 | 2 | 50 |
| 23 | f | 53 | L T | L T mesial | L F2 encephalomalacic changes | LEV (1500), LAC (200), CBZ (800), CLO (10) | L SeAH | 0.8 | 50 |
| 24 | f | 52 | bil T, L>R | L Heschl's gyrus and L post Ins | ganglioglioma adjacent to L Heschl's gyrus | CBZ (1000), LEV (2000) | L Heschl's gyrus | 1.8 | 50 |
| 25 | f | 42 | bil T, L>R | L T mesial | no lesion | LTG (350), TPM (250) | L SeAH | 3.4 | 50 |
|  |  |  |  |  |  |  |  |  |  |
| 26 | m | 40 | bil FC | no generator identified | no lesion | PHT (400), LTG (700) | no | 4.5 | 50 |
| 27 | f | 25 | bil T, R>L | bil T mesial | R T atrophy | LEV (1750), CLO (30), CBZ (1000), LAC (100) | R ant T and A | 5.3 | 50 |
|  |  |  |  |  |  |  |  |  |  |
| 28 | m | 34 | bil T | L T mesial | remote R ant T and AH resection | CBZ (1400), CLO (30) | no | 5.3 | 50 |
| 29 | m | 38 | Bil FTP, multifocal | R T neocortex > mesial; L T neocortex; L P neocortex | bil MTS | TOP (300), LTG (200), CLO (20), CBZ (1200) | no | 7.7 | 50 |
|  |  |  |  |  |  |  |  |  |  |
| 30 | m | 24 | bil T, L>R | bil T mesial, L>R | no lesion | CBZ (1600) | L SeAH | 1.4 | 36 |
|  |  |  |  |  |  |  |  |  |  |
| 31 | f | 25 | bil FT | bil T mesial | no lesion | CBZ (1200), LTG (400) | L SeAH | 5.9 | 50 |
|  |  |  |  |  |  |  |  |  |  |
| 32 | f | 31 | L T | L T mesial | no lesion | CLO (20), LEV (2000), LTG (300) | L SeAH | 6.7 | 50 |
|  |  |  |  |  |  |  |  |  |  |
| 33 | f | 25 | L T | L T mesial | L MTS | CBZ (800), LTG (600) | L ant T and AH | 0.6 | 34 |
| 34 | m | 41 | bil FC | no generator identified | no lesion | PHT (400), LTG (700) | no | 4.3 | 50 |
| 35 | m | 39 | Bil T | R T with immediate involvement of L T > L Pc, L T mesial | bil PO atrophy and gliosis; R ant T and AH resection | NA | no | 4 | 50 |
|  |  |  |  |  |  |  |  |  |  |

Legend. A, amygdala; ant, anterior; bil, bilateral; C, central; Ca, anterior cingulate gyrus; CBZ, carbamazepine; CLO, clobazam; Clon, clonazepam; f, female; F, frontal; F1, first frontal gyrus; F2, second frontal gyrus; F3, third frontal gyrus; FCD, focal cortical dysplasia; Fus, fusiform gyrus; H, hippocampus; IED, interictal epileptic discharges; inf, inferior; Ins, insula; L, left; LAC, lacosamide; LEV, levetiracetam; LTG, lamotrigine; m, man; max, maximum; MTS, mesiotemporal sclerosis; NA, not available; O, occipital; OF, orbitofrontal; OXC, oxcarbamazepine; P, parietal; Pc, precuneus; PHT, phenytoine; post, posterior; R, right; SeAH, selective amygdalohippocampectomy; SMA, supplementary motor area; sup, superior; T, temporal; TPM, topiramate.

**Figure 1.**


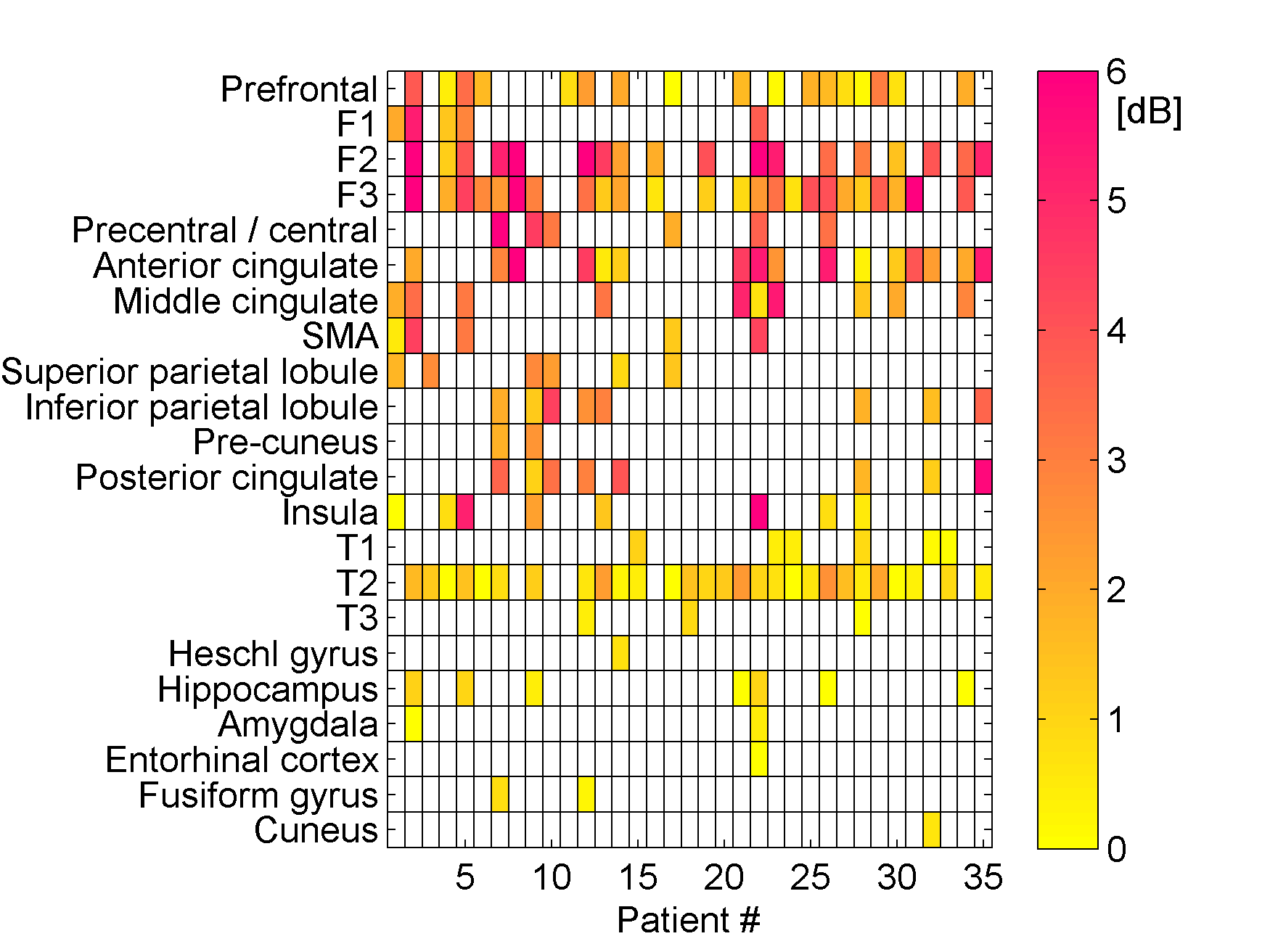


Legend. Results of sigma band energy increase at time of scalp spindles across the investigated regions of the 35 patients. The color scale is logarithmic [dB]. Note that the use of white color indicates non-investigated regions of the individual subjects.
